# A Call for Enhanced Healthcare Support for Increasingly Vulnerable Groups of Foreigners in Japan: Insights from a Former Thai-Native Medical Student in Japan

**DOI:** 10.21315/mjms2021.28.5.16

**Published:** 2021-10-26

**Authors:** Sopak Supakul, Akihiko Ozaki, Tetsuya Tanimoto

**Affiliations:** 1 Medical Governance Research Institute, Minato City, Tokyo, Japan; 2 Department of Breast Surgery, Jyoban Hospital of Tokiwa Foundation, Iwaki, Fukushima, Japan

Dear Editor,

Despite substantial global improvement in overall population health, it has been well documented that the health status of some groups of minorities is worse than that of ethnic majority groups in many countries. This is particularly relevant amidst the ongoing novel coronavirus (COVID-19) pandemic, which is disproportionately affecting the socially vulnerable population (1). While these phenomena have been prominent in countries such as the United States and Europe (2), countries that have accepted higher numbers of tourists and immigrants are experiencing a similar situation and Japan is no exception. Under former prime minister Shinzo Abe (2012–2020) in particular, visa exemptions for many nationalities with respect to short-term stays, followed by the launch of the Technical Intern Training Program attracted a large number of tourists and workers to Japan (3). According to the Japan National Tourism Organization, the number of foreign visitors to Japan in 2019 was 31,882,049, compared to 8,358,105 in 2012 (4). Among these, the number of registered foreign workers increased from 682,000 in 2012 to 1,659,000 in 2019, with the number of people who remained in the country illegally increasing from 67,065 to 74,167 in the same years (5). In this context, the health and well-being of foreigners residing in Japan is an emerging concern, given that healthcare services for foreigners can become complicated due to multifaceted barriers to access, such as poor language abilities, cultural norms and financial struggles (6–7). Notably, these issues have already caused critical problems for both care providers and care receivers in Japan.

As a Thai national who attended medical school in Japan, the lead author of this paper (SS), together with Thai embassy staff, has been involved in multiple cases related to healthcare support for Thai nationals in Japan. Among them was an uninsured Thai tourist with acute myocardial infarction who underwent surgeries at a university hospital. Given that the patient had no travel insurance, the medical team and the patient’s family initially discussed the decision to perform surgery intensively, with SS as an interpreter. Both the patient and her family encountered multiple communication and funding problems, but they eventually received financial support from the Thai government and other support, including communication, from the local embassy staff and a group of international students. Similar situations occur among foreign workers, as illustrated in another case involving an illegal Thai worker who was struck by lightning, resuscitated and later remained in a vegetative state. The CT scan and electroencephalogram confirmed brain death but vital signs were present. Although the family could not afford the hospital fees, they wished to prolong the patient’s life and perform a religious ritual seeking a miracle. The family’s desire added the complication of the intercultural context to the family’s financial problems. As a Thai-native, SS, who was assisting as an interpreter, was able to explain the religious premise behind the family’s request to the Japanese medical staff. Together, these two cases suggest that the barriers vulnerable groups of foreigners often encounter with regard to medical services can be mainly divided into financial strain and communication problems associated with linguistic and cultural norms.

In Japan, long-term foreign residents who are staying in the country legally have the right to enroll in the national insurance system, which provides access to universal health coverage, meaning that, like Japanese citizens, their medical fees are covered when they use medical services. However, although some minority groups, such as uninsured tourists and illegal workers, can receive medical care in emergency situations, their medical fees are not covered under the national insurance system. As the number of foreigners in Japan increases annually, the number of medical cases in which the medical bills remain unpaid is also increasing. This could put pressure and stress on the physicians and other hospital staff who deal with such foreigners (7). Other than requesting help from the patient’s relatives in their home country and/or the appropriate embassy, there is currently no proper system for seeking assistance and nonpayment has shifted the financial burden onto hospitals. Thus, these cases are becoming problematic for both care receivers and care providers. Regarding tourists, the ideal solution would be a regulation requiring that all foreigners entering Japan show proof of travel insurance. In addition, raising global awareness about the importance of securing medical travel insurance prior to travel is crucial. Furthermore, it is important to have a system that identifies illegal workers and repatriates them before they fall ill; this is a function that Japan’s immigration bureau already performs. However, in cases where medical services have been provided, an efficient tracking system to collect medical fees from patients who have returned to their home country without paying their bill is urgently needed.

Regarding communication issues, SS’s presence as an interpreter in the cases described above was a mere coincidence that helped the patients access the support they needed and allowed the medical staff to work more smoothly. However, increasing the number of health workers from overseas is quite impractical and unlikely to solve such a pervasive problem, given that Japan has always and will continue to be an unattractive destination for international medical students and graduates because of the relatively low salaries compared to other developed countries and the language barrier, as well as the Japanese cultural preference for domestic healthcare professionals. While the number of doctors in Japan was 340,964 in 2014 (8), it is speculated that the number of non-Japanese holders of a Japanese medical license is extremely low. One survey from the Japan Hospital Association in 2015 showed that among the 669 Japanese hospitals participating in the survey, there were only 17 non-Japanese doctors (9). Moreover, among these hospitals, 77.3% had never accepted international healthcare workers and, surprisingly, 77% of them showed no interest in doing so. Compared to the United States, where physicians from overseas account for approximately one-quarter of the total number (10) and the United Kingdom, where international doctors comprise one-third (11), the number of internationally-trained non-Japanese physicians in Japan is far behind. As such, increasing the number of international doctors seems unrealistic. Regarding the global perspective on the relevant issues, in the United States, the Emergency Medical Treatment and Labor Act (EMTALA) has stipulated that hospitals must treat every emergency room patient, regardless of their ability to pay; this includes undocumented immigrants. In addition, a survey of 4,586 hospitals conducted in the United States in 2016 showed that only 56% offered linguistic services for patients who have difficulty speaking English (12). While the United States is well known for its ethnically diverse population, providing care for foreign patients is still a problem.

In Japan, local medical staff have made some efforts to improve the situation by, for example, encouraging healthcare workers to enroll in medical translation courses, hiring professional medical interpreters and coordinators at many hospitals and adding more medical English classes to many medical schools’ curricula. Although it would be ideal to have more foreign medical workers taking care of foreign patients, the local staff’s attempts to improve the personal skills and perspectives that are needed to care for foreigners are also crucial. Indeed, these changes have reflected a gradually increasing awareness of the growing number of foreign patients in the Japanese healthcare sector. Although it is still unclear whether vulnerable groups of foreigners residing in urban and rural areas face different barriers and receive different care, urban–rural discrepancies might exist and should be further elucidated (13). At present, there is an ongoing trend in which a growing number of hospitals in rural areas of Japan accept nurses and care providers from overseas to compensate for the medical staff shortage; this is the case at the hospital where the second author (AO) works (14). We believe that although there are multiple obstacles involved, these attempts constitute a possible measure to mitigate the challenging circumstances affecting vulnerable groups of foreigners (14).

Presently, as many countries’ governments are focusing on accepting more foreign workers, as well as tourists, to maintain economic growth, the demand for healthcare support for foreigners is also increasing, although, as of 2020, the trend has been temporarily deterred by the COVID-19 pandemic (15). However, as it is expected that the number of tourists and workers will gradually recover after the pandemic ends, we believe that every government should prepare countermeasures to address this issue. Although it is desirable for foreigners to protect their own health, there should be an increasing awareness of the necessity of policy-level interventions to help safeguard their health and well-being, not only in Japan but also in other countries.
